# Risk factors for procedure-related complications after endoscopic resection of colorectal laterally spreading tumors

**DOI:** 10.1097/MD.0000000000012589

**Published:** 2018-10-12

**Authors:** Ji-Yun Hong, Sun-Seog Kweon, Jun Lee, Sang-Wook Kim, Geom-Seog Seo, Hyun-Soo Kim, Young-Eun Joo

**Affiliations:** a Department of Internal Medicine; b Department of Preventive Medicine, Chonnam National University Medical School; c Department of Internal Medicine, Chosun University College of Medicine, Gwangju; d Department of Internal Medicine, Chonbuk National University Medical School, Jeonju; e Department of Internal Medicine, Wonkwang University College of Medicine, Iksan, Korea.

**Keywords:** bleeding, endoscopic resection, laterally spreading tumor, perforation, risk factors

## Abstract

Colorectal laterally spreading tumors (LSTs) are large and flat elevated neoplasms with diameters of at least 10 mm. Endoscopic resection of LSTs, with their large size and broad base, is difficult and dangerous compared with the resection of polypoid neoplasms. This study aimed to determine the risk factors for procedure-related complications including bleeding and perforation after endoscopic resection of LSTs.

Patients with colorectal LST undergoing endoscopic resection at 5 university hospitals in Honam Province of South Korea were enrolled, and their records about patients, lesions, and procedure parameters associated with the occurrence of complications were reviewed retrospectively. Logistic regression analysis was performed to identify risk factors for complications.

The frequency of comorbidities in bleeding group was significantly higher than in the no bleeding group. The frequency of bleeding was significantly higher in lesions with adenocarcinoma than in lesions with low or high-grade dysplasia. The frequency of bleeding was significantly higher in piecemeal resection than in en bloc resection. The frequency of perforation was significantly higher in endoscopic mucosal resection-precutting (EMR-P) than in endoscopic mucosal resection (EMR) or endoscopic submucosal dissection. The mean procedure duration was significantly longer in the perforation group than in the no perforation group. On multivariate analysis, patient comorbidity and histologic grade of the lesion were significant independent risk factors for bleeding, whereas EMR-P was a significant independent risk factor for perforation after endoscopic resection.

This study demonstrated that patient comorbidity and histologic grade of lesion were significant independent risk factors for bleeding, and EMR-P was a significant independent risk factor for perforation after endoscopic resection of colorectal LSTs.

## 1 Introduction

Colorectal laterally spreading tumors (LSTs) are large and flat precancerous lesions with a diameter of at least 10 mm that extend laterally along the luminal wall with a low vertical axis.^[1–4]^ Colorectal LSTs are now increasingly reported due to increased awareness, the wider application of screening colonoscopy, and optical enhancement technologies such as chromo and magnifying colonoscopy.^[1–4]^ Colorectal LSTs have been found in about 1.3% of asymptomatic patients who underwent screening colonoscopy, and they represent 17.2% of advanced neoplasia.^[3]^ Colorectal LSTs are classified into the granular type, including homogeneous and nodular mixed subtypes, and the nongranular type, including flat elevated and pseudo-depressed subtypes based on their detailed endoscopic macroscopic appearance.^[4]^

Endoscopic resection including endoscopic mucosal resection (EMR) and endoscopic submucosal dissection (ESD) are widely accepted treatments for superficial colorectal neoplasms. While endoscopic resection of colorectal neoplasms has been proven feasible and safe, it is associated with a small but definitive incidence of procedure-related complications such as bleeding and perforation. Several studies have evaluated the risk factors predictive of complications after endoscopic resection.^[5–11]^ Also, the identification of such risk factors may provide important and useful information for endoscopists to consider before procedures.

The frequency of submucosal invasive carcinoma in colorectal LSTs is lower than that in polypoid lesions of a similar diameter.^[12–17]^ Therefore, colorectal LSTs are primarily treated by EMR and ESD.^[18–27]^ However, endoscopic resection of colorectal LSTs typically represents a technical challenge for endoscopists in terms of the ability to achieve complete resection without complications, because colorectal LSTs are flat neoplastic lesions with a large size and broad base.

In this study, we retrospectively evaluated the data on endoscopic resection for colorectal LSTs and sought to determine the risk factors associated with procedure-related complications such as bleeding and perforation.

## 2 Materials and methods

### 2.1 Study population

This retrospective, multicenter cohort study included patients who underwent endoscopic resection of colorectal LSTs between January, 2012 and December, 2013 at 5 university hospitals in the Honam Province of South Korea that are affiliated with the Honam Association for Study of Intestinal Diseases (HASID). This study protocol was approved by the ethical review boards of all participating institutions, and written informed consent was obtained from all patients before the endoscopic resection procedure. One physician at each institution was responsible for data collection, and the completeness of the data collection was monitored by 1 of the authors (Y.E.J.). In all, 841 patients with colorectal LSTs who underwent endoscopic resection such as EMR, EMR-precutting (EMR-P), and ESD were reviewed. We excluded 247 patients due to a lack of complete clinicopathological data (197 patients) and the presence of non-neoplastic lesions such as hyperplastic polyp and chronic colitis (50 patients). Finally, a total of 594 patients were analyzed.

### 2.2 Endoscopic features of colorectal LSTs

All patients were examined using video-colonoscopes (CF-240I or CF-Q260AI; Olympus Optical Co., Tokyo, Japan). LSTs are classified into 2 types: the granular type, including homogeneous and nodular mixed subtypes; and the nongranular type, including flat elevated and pseudo-depressed subtypes.^[4]^ The pit pattern was divided into 7 types according to the Kudo classification system: the non-neoplastic (type I/II), adenomatous (type IIIs/IIIL/IV), and cancerous types (type Vi/Vn).^[28]^ The pit pattern of the LSTs was evaluated retrospectively by 2 observers (J.Y.H. and Y.E.J.) via analysis of conventional colonoscopy, NBI, or chromoendoscopic images. Among the 594 LSTs, a consensus was reached for 445 LSTs by interobserver agreement. The location of the LSTs was divided into the distal colon (rectum, sigmoid colon, and descending colon) and the proximal colon (cecum, ascending colon, and transverse colon). The size of each lesion was determined endoscopically by comparison with the diameter of the snare.

### 2.3 Endoscopic procedures

Endoscopic procedures including EMR, EMR-P, and ESD were performed by experienced endoscopists who had previously performed more than 1000 therapeutic endoscopic procedures. The endoscopic treatment modality was selected by each endoscopist based on their experience and preference. Endoscopic procedures were performed using Olympus endoscopes (CF-240I or CF-Q260AI) in all participating institutions. To delineate the margins of the lesions and evaluate the pit patterns, chromoendoscopy using 0.4% indigocarmine dye or narrow band imaging was performed. An ERBE ICC 200 or VIO-300 D electrocautery device (ERBE Electromedizin, Tübingen, Germany) was used for cutting and coagulation.

#### 2.3.1 EMR technique

Endoscopic mucosal resection was performed using the ‘lift and cut’ technique. The mixture of normal saline and indigocarmine with diluted epinephrine (1:5000-1:10000) was injected into the submucosal layer below the lesion using a 23-gauge needle (NM-4U-1; Olympus) until the mucosa was lifted. The lifted lesion was then excised by constriction and electrical current using a snare wire (SD-12L/U-1; Olympus) and an electrocautery device (ERBE). In cases of piecemeal resection, all pieces of the lesion were collected with retrieval devices including a tripod, pentapod, and net. If there was suspicion of remnant lesion after piecemeal resection, argon plasma coagulation was applied.

#### 2.3.2 EMR-P technique

Endoscopic mucosal resection-precutting was performed by injection of the mixture solution into the submuocosal layer and the circumferential incision of the mucosa with flex knife (Olympus). After additional injection of the mixture solution beneath the lesion, we applied a snare (SD-9U-1 or SD-12U-1; Olympus) around the tumor at the mucosal incision site and removed the lesion in the same fashion as with the standard snare polypectomy technique.

#### 2.3.3 ESD technique

Endoscopic submucosal dissection was performed using a dual knife (Olympus), a flex knife (Olympus), and a flush knife (Olympus) through a standard, single-channel endoscope (Olympus CFQ260AI). The mixture solution was injected into the submucosal layer, and a circumferential mucosal incision was made using a flush knife or dual knife. Then, the submucosal layer was dissected using a flush knife, dual knife, or flex knife in the Endo-Cut mode (Effect 3, 60–80 W). Hemorrhage was controlled using hemostatic forceps, such as the Coagrasper (Olympus) in the soft coagulation mode (50 W). All procedures were performed by endoscopists who had each performed more than 100 colorectal ESD procedures. Finally, the resected specimen was retrieved with grasping forceps.

### 2.4 Histopathological assessment of colorectal LSTs

For histopathological assessment, all resected specimens were stretched and pinned out, immediately fixed in a 10% buffered formalin solution, and examined histologically using hematoxylin and eosin staining. All resected specimens were examined by experienced gastrointestinal pathologists. Histopathological diagnosis was performed according to the World Health Organization classification system.^[29]^

### 2.5 Definition of resection type, procedure duration, and complete resection

En bloc and piecemeal resections were defined as a specimen removed in a single piece and multiple pieces, respectively. Procedure duration was counted from the beginning of submucosal injection to the completion of the removal of the lesion. Complete resection (R0) was confirmed if both the lateral and vertical margins were free of tumor cells.

### 2.6 Definition of complications

Postprocedural bleeding was defined as the clinical evidence of hemorrhage with melena or hematochezia, decrease in hemoglobin of >2 g/dL, requirement of blood transfusion, or the requirement of postprocedural endoscopic hemostasis. Perforation was defined as the endoscopic finding of mesenteric fat or intra-abdominal space noted during procedure or the presence of free air on plain radiography or computed tomography after procedure.

### 2.7 Statistical analysis

Statistical analyses were performed using the Statistical Package for the Social Sciences (Version 18.0; SPSS, Chicago, IL). Descriptive analyses included proportions for categorized data and means for continuous data. Risk factors for bleeding and perforation were determined using a logistic regression model. All risk factors were analyzed by univariate logistic regression analysis, and factors with a *P* value <.05 were included in the multivariate logistic regression model. A *P* value of <.05 was considered statistically significant.

## 3 Results

### 3.1 Baseline characteristics of patients with colorectal LSTs treated by endoscopic procedures

The baseline characteristics of patients with LSTs treated by endoscopic procedures are summarized in Table 1. The mean age of the patients was 65.6 ± 9.8 years (range 39–90). This study group comprised 377 males (63.5%) and 217 females (36.5%). In all, 339 patients (57.1%) had comorbidities such as cardiovascular, cerebrovascular, chronic liver, or chronic renal disease. Also, 151 (25.4%) and 177 (29.8%) patients had a history of smoking and alcohol consumption, respectively. Medications including aspirin and nonsteroidal anti-inflammatory drugs (NSAIDs) were used in 89 patients (15.0%). The mean size of the LSTs was 24.2 ± 11.3 mm (range 10.0–70.0). A total of 319 LSTs (53.7%) were localized in the distal colon and 275 (46.3%) were localized in the proximal colon. Among 594 LSTs, 394 (66.3%) were granular-type LSTs, including 144 homogenous (24.2%) and 250 nodular mixed subtypes (42.1%), whereas 200 (33.7%) were nongranular type LSTs, with 161 flat elevated (27.1%) and 39 pseudo-depressed subtypes (6.6%). In the analysis of pit patterns, 70 (11.8%) lesions were type I, 24 (4.0%) were type II, 100 (16.8%) were type IIIs, 169 (28.5%) were IIIL, 20 (3.4%) were IV, 40 (6.7%) were Vi, and 22 (3.7%) were Vn. Histopathological diagnosis revealed 348 (58.6%) low-grade dysplasias, 110 (18.5%) high-grade dysplasias, and 136 (22.9%) adenocarcinomas. The LSTs were removed by EMR (294, 49.5%), EMR-P (91, 15.3%), and ESD (209, 35.2%). The en bloc and piecemeal resection rates were 83.5% (496/594) and 16.5% (98/594), respectively. The mean procedure duration was 30.6 ± 39.8 minutes (range 1.0–330.0). The complete resection (R0) rate was 89.1% (529/594). The postprocedural bleeding rate was 7.4% (44/594) and the perforation rate was 2.0% (12/594).

**Table 1:**
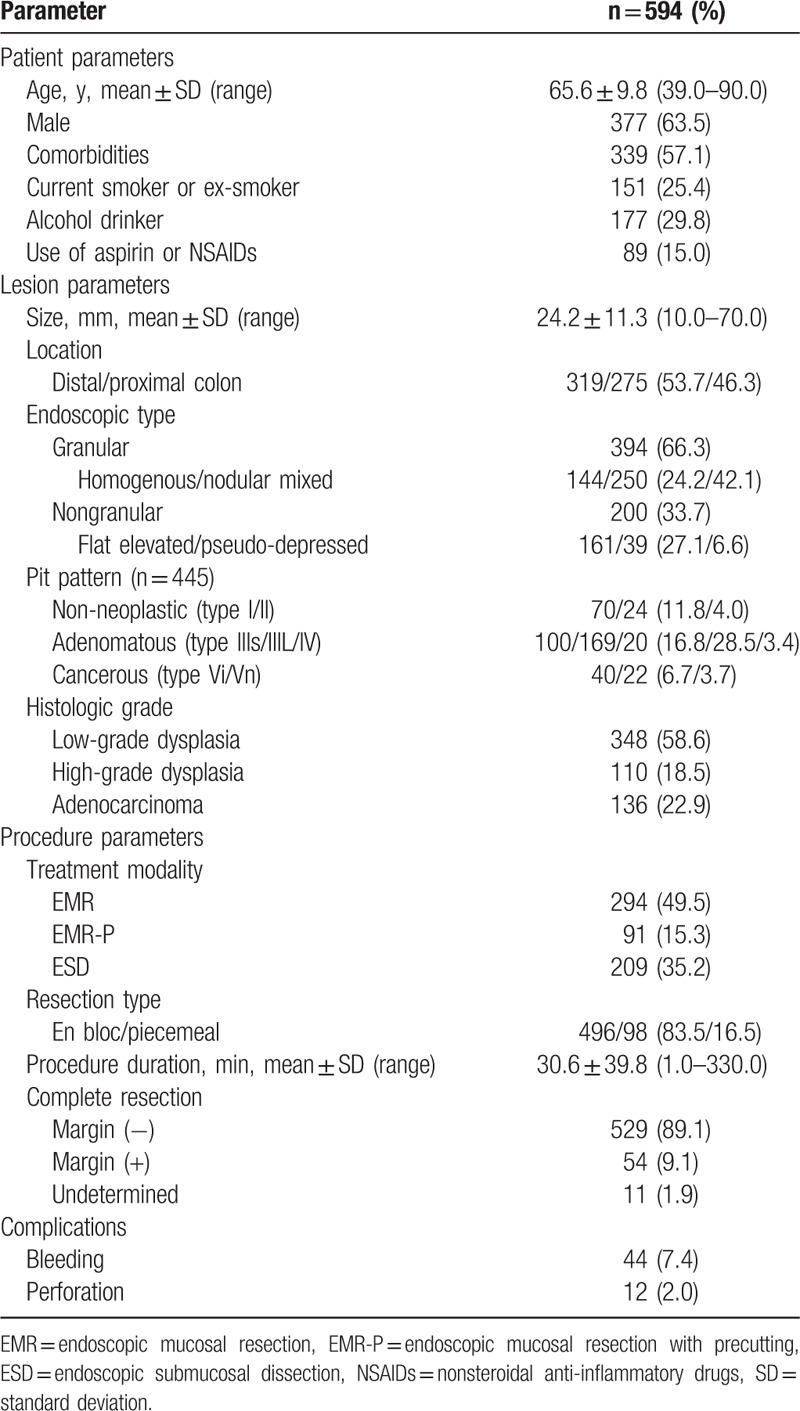
Baseline characteristics of patients with colorectal laterally spreading tumors treated by endoscopic procedures.

### 3.2 Analysis of risk factors for bleeding after endoscopic resection of colorectal LSTs

The clinicopathological characteristics of the cases without or with bleeding are summarized in Table 2. With regard to the patient parameters, the frequency of comorbidities was significantly higher in the bleeding group than in the no-bleeding group (odds ratio [OR] 2.111, 95% confidence interval [CI] 1.065–4.185, *P* = .032). There was no significant difference between the groups with and without bleeding in terms of age, sex, smoking, alcohol, and current use of aspirin or NSAIDs. With regard to the lesion parameters, the frequency of bleeding was significantly higher in lesions with adenocarcinoma than in lesions with low or high-grade dysplasia (OR 2.985, 95% CI 1.539–5.791, *P* = .001). There was no significant difference between the groups with and without bleeding in terms of the size, location, endoscopic features, and pit patterns of the lesions. With regard to the procedure parameters, the frequency of bleeding was significantly higher in piecemeal resection than in en bloc resection (OR 2.023, 95% CI 1.002–4.083, *P* = .049). There was no significant difference between the groups with and without bleeding in terms of treatment modality and procedure duration.

**Table 2:**
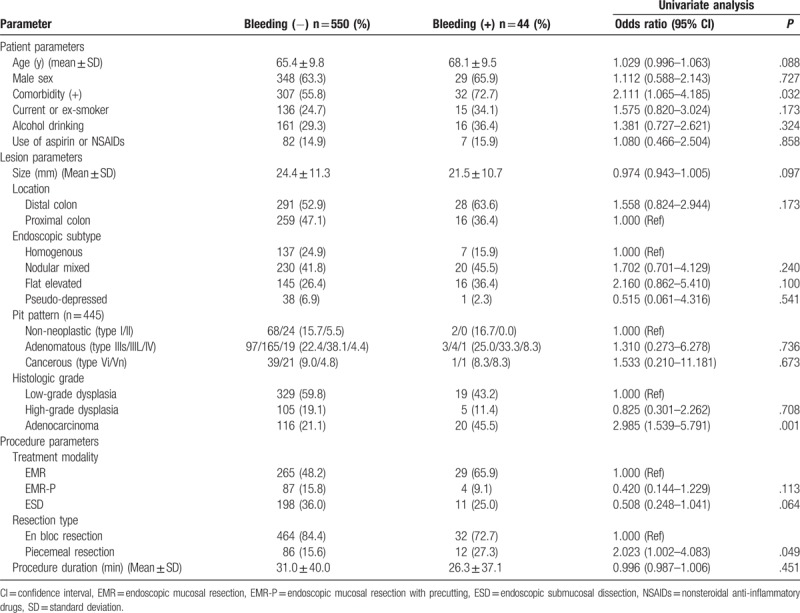
Analysis of risk factors for bleeding after endoscopic treatment of colorectal laterally spreading tumors.

### 3.3 Analysis of risk factors for perforation after endoscopic resection of colorectal LSTs

The clinicopathological characteristics of the cases with or without perforation are summarized in Table 3. With regard to the patient parameters, there was no significant difference between the groups with and without perforation in terms of age, sex, comorbidity, smoking, alcohol, and current use of aspirin or NSAIDs. With regard to the lesion parameters, there was no significant difference between the groups with and without perforation in terms of size, location, endoscopic features, pit patterns, and histologic grade. With regard to the procedure parameters, the frequency of perforation was significantly higher in EMR-P than in EMR or ESD (OR 20.682, 95% CI 2.456–174.166, *P* = .005). The mean procedure duration was significantly longer in the perforation group than in the no-perforation group (OR 1.012, 95% CI 1.004–1.019, *P* = .002). There was no significant difference between the groups with and without perforation in terms of the resection type.

**Table 3:**
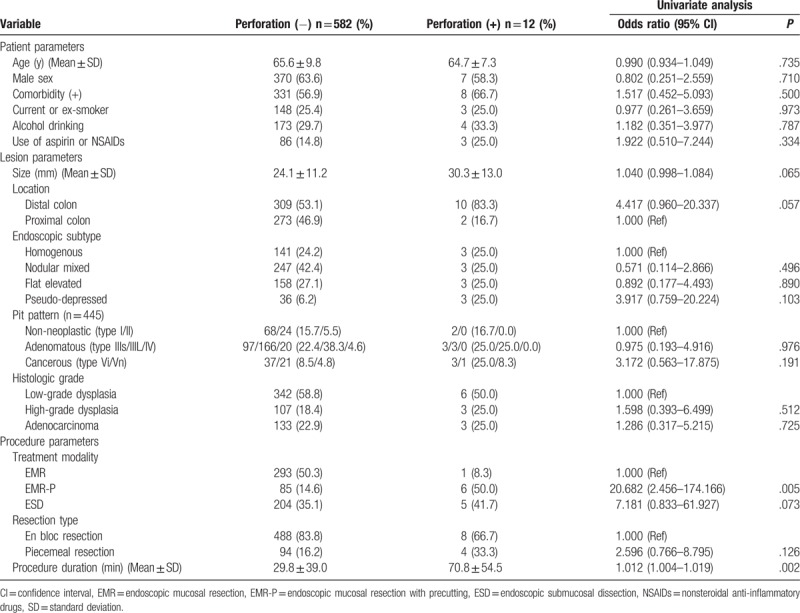
Analysis of risk factors for perforation after endoscopic treatment of colorectal laterally spreading tumors.

### 3.4 Multivariate analysis of risk factors for bleeding and perforation after endoscopic resection of colorectal LSTs

On multivariate logistic regression analysis, the comorbidity and histologic grade of the lesions were significant independent risk factors for bleeding after endoscopic resection (OR 2.058, 95% CI 1.023–4.098, *P* = .04 and OR 2.589, 95% CI 1.309–5.119, *P* = .006, respectively), whereas the EMR-P and procedure duration were significant independent risk factors for perforation after endoscopic resection statistically (OR 18.166, 95% CI 2.151–153.438, *P* = .008 and OR 1.014, 95% CI 1.004–1.023, *P* = .005, respectively) (Table 4).

**Table 4:**
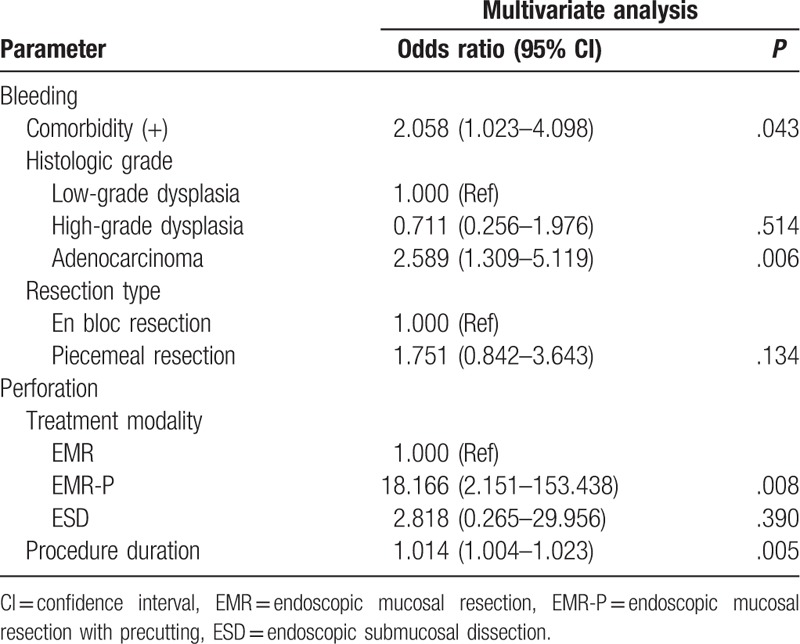
Multivariate analysis of risk factors for complication after endoscopic treatment of colorectal laterally spreading tumors.

## 4 Discussion

Colorectal LSTs are the superficial flat elevated precancerous lesions with diameters of at least 10 mm.^[1–4]^ Endoscopic resection, including EMR and ESD, is a safe and effective treatment for precancerous and early colorectal neoplasia.^[5–11]^ Currently, most LSTs are treated by endoscopic resection to avoid increased surgical risk and potentially significant mortality, morbidity, and cost. As shown by many previous reports, endoscopic resection is successful in 70% to 100% of cases.^[18–27]^ Similarly, in our study, the complete resection (R0) rate was 89.1%.

Bleeding and perforation are the major complications associated with endoscopic resection.^[5–11]^ In particular, the endoscopic resection of LSTs, with their larger size and broader base, is difficult and dangerous compared with the resection of polypoid neoplasms.^[18–27]^ The aim of our study was to evaluate the patient, lesion, and procedure parameters associated with the occurrence of procedure-related complications such as bleeding and perforation.

Bleeding is the most common complication after endoscopic resection. Bleeding associated with EMR and ESD has a wide-ranging incidence of 1.0% to 18.0% and 0% to 15.6%, respectively.^[5–11]^ This is likely to be due to variability in the definitions of bleeding across different reports. In our study, the overall postprocedural bleeding rate was 7.4%, which is in accordance with other reports. Generally, factors affecting the risk of postprocedural bleeding include lesion size, flat morphology, location, patient comorbidities, coagulation status, and lesion histology after endoscopic resection.^[5–11]^ However, the reported risk factors are inconsistent across different reports.

In the present study, we analyzed the risk factors for bleeding after endoscopic resection, and postprocedural bleeding was significantly associated with patient comorbidity, lesions with adenocarcinoma, and piecemeal resection. The patients with comorbidities such as cardiovascular, cerebrovascular, chronic liver, or chronic renal disease typically have some degree of coagulopathy due to the disease itself or to medications such as warfarin.^[8]^ In terms of lesions with adenocarcinoma, postprocedural bleeding may occur more frequently due to rapid growth and increased neovascularization toward the submucosa. Moreover, piecemeal resection may lead to increased risk of bleeding and perforation owing to the repeat application of electrical current and inadequate submucosal injection for each resection. Based on multivariate analysis, patient comorbidity and the histologic grade of the lesion were found to be significantly and independently associated with bleeding after endoscopic resection.

Perforation is the most serious complication of endoscopic resection because of the possibility of subsequent peritonitis. EMR, EMR-P, and ESD-related perforation rates were reported as 0.09% to 3.1%, 2.9% to 8.8%, and 1.4% to 10.4%, respectively.^[5–11]^ In our study, perforation rate of EMR-P was 6.6%, which is in accordance with previous reports, but higher than that of EMR (0.3%) or ESD (2.4%), although all of the endoscopic procedures were performed by same highly experienced endoscopists. Overall perforation risk is largely influenced by older age, concurrent comorbidities, lesion size, sessile morphology, right-sided location, the choice of snare or resection tool, application of electrocautery and settings, inadequacy of submucosal cushion for removal of larger lesions, submucosal fibrosis, and endoscopist inexperience.^[5–11]^

Next, we analyzed the risk factors for perforation after endoscopic resection. Our finding showed perforation was significantly associated with EMR-P and procedure duration. Moreover, lesion size tended to be larger in the perforation group than in the nonperforation group. EMR-P was primarily used for the resection of colorectal LSTs, for which en bloc resection by EMR cannot be performed due to their large size and difficult location.^[9]^ Furthermore, if snaring and resection of the lesion is performed without adequate additional submucosal injection beneath the lesion after the circumferential mucosal incision during EMR-P, it may lead to an increased risk of perforation. Procedure duration in the perforation group was significantly longer than in the nonperforation group statistically. The multivariate analysis showed that procedure duration was a significant risk factor for perforation, but we thought that it was not clinically relevant because of very small odds ratio (OR 1.014, 95% CI 1.004–1.023). Fatigue of the endoscopist due to prolonged procedure contributed to the occurrence of perforation, but the occurrence of perforation itself was a factor for prolonging the procedure duration, so they were not completely independent factors for each other.

This study has some limitations. Resulting from its retrospective and multicenter design, there were some important possible factors that not be fully investigated due to lack of records such as degree of submucosal fibrosis, location of the lesions (especially whether they located in hepatic or splenic flexure that are difficult to resect), and the accessories used for resection like knives, and so on. Second, the number of cases with complications such as bleeding and perforation in each treatment modality was relatively small. Further larger, prospective studies are needed to clarify the risk factors associated with complications in each modality.

## 5 Conclusions

In conclusion, this study demonstrated that the patient comorbidity and histologic grade were significant independent risk factors for bleeding, and that EMR-P was a significant independent risk factor for perforation after the endoscopic resection of colorectal LSTs. These results may help to inform the endoscopic management of colorectal LSTs.

## Author contributions

**Conceptualization:** Young-Eun Joo.

**Data curation:** Sun-Seog Kweon, Jun Lee, Sang-Wook Kim, Geom-Seog Seo, Hyun-Soo Kim.

**Formal analysis:** Sun-Seog Kweon, Jun Lee, Sang-Wook Kim, Geom-Seog Seo, Hyun-Soo Kim.

**Investigation:** Ji-Yun Hong, Young-Eun Joo.

**Methodology:** Young-Eun Joo.

**Project administration:** Young-Eun Joo.

**Resources:** Sun-Seog Kweon, Jun Lee, Sang-Wook Kim, Geom-Seog Seo, Hyun-Soo Kim.

**Writing – original draft:** Young-Eun Joo.

**Writing – review & editing:** Ji-Yun Hong, Young-Eun Joo.
